# Increased risk of cardiomyopathy in individuals with methamphetamine related disorders in Taiwan

**DOI:** 10.1038/s41598-025-94591-0

**Published:** 2025-04-03

**Authors:** Pi-Ching Yu, Ho-Tsung Hsin, Wei-Ting Lin, Yao-Ching Huang, Shi-Hao Huang, Tsu-Hsuan Weng, Bing‑Long Wang, Chi-Hsiang Chung, Li-Yun Fann, Wu-Chien Chien, Sung-Sen Yang

**Affiliations:** 1 Graduate Institute of Medicine, National Defense Medical Center, Taipei, 11490 Taiwan; 2 Cardiovascular Intensive Care Unit, Department of Critical Care Medicine, Far-Eastern Memorial Hospital, New Taipei City, 10602 Taiwan; 3 Department of Education, Taipei City Hospital, Taipei, 10684 Taiwan; 4 Department of Chemical Engineering and Biotechnology, National Taipei University of Technology (Taipei Tech), Taipei, 10608 Taiwan; 5 Department of Medical Research, Tri-Service General Hospital, Taipei, 11490 Taiwan; 6 School of Public Health, National Defense Medical Center, Taipei, 11490 Taiwan; 7 Department of Health Administration, Asia University, Taichung, 41354 Taiwan; 8 Taiwanese Injury Prevention and Safety Promotion Association (TIPSPA), Taipei, 11490 Taiwan; 9 Department of Nursing, Taipei City Hospital, Taipei, 10684 Taiwan; 10 Graduate Institute of Life Sciences, National Defense Medical Center, Taipei, 11490 Taiwan; 11 Department of Nephrology, Tri-Service General Hospital, Taipei, 11490 Taiwan

**Keywords:** Methamphetamine-related disorders (MRDs), Non-methamphetamine—related disorders (non-MRDs), Cardiomyopathy, Longitudinal generation tracking database (LGTD), Biological techniques, Drug discovery, Evolution, Physiology, Cardiology, Diseases, Health care, Medical research, Risk factors

## Abstract

To explore whether Methamphetamine-related disorders (MRDs) will cause the risk of cardiomyopathy in the future. This study used Taiwan’s Longitudinal Generation Tracking Database (LGTD) to conduct a 1:4 paired analysis of sex, age, and inclusion year. 17,071 patients with MRDs and 153 patients with cardiomyopathy were selected; 68,264 patients without MRDs and 274 patients with cardiomyopathy were also selected. This study used SPSS 22 statistical software to conduct Cox regression analysis. Patients with MRDs had a 3.421-folds higher risk of cardiomyopathy than patients without MRDs. Men have a 0.735-fold lower risk of developing cardiomyopathy than women. In terms of age group, aged 50–64 and ≧ 65 have a 1.145- and 1.332-folds higher risk of cardiomyopathy, respectively, compared to those aged 20–49. For each one-point increase in Charlson Comorbidity Index (CCI), the risk of cardiomyopathy rises by 58.3%. Specifically, for three types of Methamphetamines (Methamphetamine and other psychostimulant dependence, Methamphetamine or related acting sympathomimetic abuse, Methamphetamine psychosis), the HR for cardiomyopathy in patients with MRDs was 3.864 (*p* < 0.001), 2.916 (*p* < 0.001), and 2.295 (*p* = 0.016) times higher, respectively, compared to patients without MRDs. The Kaplan-Meier log-rank test was used to calculate the cumulative risk of MRDs, showing a significant difference in the cumulative cardiomyopathy incidence between the MRDs and non-MRDs groups (long-rank test, *p* < 0.001). MRDs will increase the risk of cardiomyopathy. Women are more susceptible to cardiomyopathy than men, and the risk escalates for individuals aged 50–64 and those 65 years or older, compared to the 20–49-year age group. Additionally, an increase in the CCI correlates with a heightened risk of cardiomyopathy. There are important differences between these groups in terms of duration, frequency, and severity of use, with longer exposure and more frequent use increasing the risk of dependence and psychosis, but individual susceptibility, dose, and use patterns also play key roles.

**Supplementary Information:**

The online version contains supplementary material available at 10.1038/s41598-025-94591-0.

## Introduction

Amphetamines were first synthesized in the late 1920s as an analogue of the popular drug ephedrine^1^. These drugs are mainly used for nasal congestion relief, asthma, narcolepsy, depression, and weight loss, and less frequently for heart block, myasthenia gravis, dysmenorrhea, and persistent hiccups^2^. Amphetamine patents in the 1920s forced competitors to synthesize methamphetamine, which was used for many of the same indications as amphetamine^3^. However, recognition of their serious addictive potential led to production restrictions, but the drugs entered the black market, thus starting an epidemic. The estimated prevalence of illicit drug use among people aged 12 years and older in the United States was 13.0% in 2019, and 0.6% (approximately 1.5 million people) of people aged 12 years and older reported having methamphetamine use disorder in 2020^4,5^. The average duration of methamphetamine use before a diagnosis of congestive heart failure was five years, with almost half (18%) of reported diagnoses made within the previous 12 months. In some cases, congestive heart failure was diagnosed even after a single use^6^. Methamphetamine use induces potent vasoconstriction, resulting in severe vasospasm of the coronary arteries and microvasculature, resulting in myocardial ischemia. In the heart, methamphetamine promotes myocardial structural and electrical remodeling, which may promote arrhythmias. Ultimately, methamphetamine induces severe mitochondrial dysfunction and cardiomyocyte death, leading to dilated cardiomyopathy and heart failure^7,8^. Amphetamine use causes cardiomyopathy through catecholamine-mediated effects such as tachycardia, hypertension, vasoconstriction, and direct cardiotoxic effects^9,10^.

Cardiomyopathy results from changes in the structure and function of the myocardium caused by a variety of potential causes, which can be localized to the heart or cause systemic disease^11^. Cardiomyopathy can be classified as primary (e.g., hereditary, mixed, or acquired) or secondary (e.g., infiltrative, toxic, inflammatory). The main types include dilated cardiomyopathy, hypertrophic cardiomyopathy, restrictive cardiomyopathy and arrhythmogenic right ventricular cardiomyopathy^11^. Hypertrophic cardiomyopathy is the most common primary cardiomyopathy and can cause active dyspnea, syncope, atypical chest pain, HF, and sudden cardiac death. Dilated cardiomyopathy can be hereditary or acquired and usually presents with classic symptoms of HF with reduced ejection fraction of the heart. Restrictive cardiomyopathy is less common and is usually associated with systemic disease^12^.

Cardiomyopathy is a heterogeneous disease and a leading cause of heart failure (HF) and is associated with significant morbidity and mortality^13^. HF often coexists with many comorbidities, among which decreased renal function is particularly important. The interaction between the heart and kidneys is complex and bidirectional^14^, and 75% of patients have left ventricular hypertrophy at the start of dialysis^15^.

There are currently limited studies on the relationship between Methamphetamine-related disorders (MRDs) and cardiomyopathy. Therefore, we hypothesized that MRDs and cardiomyopathy have related risk effects, and we used Taiwan’s Longitudinal Generation Tracking Database (LGTD) from 2000 to 2015 to analyze the impact of MRDs and cardiomyopathy through long-term follow-up.

## Materials and methods

### Data source

Since 1995, Taiwan’s Ministry of Health and Welfare has maintained the National Health Insurance Research Database (NHIRD), This study utilized data from the Longitudinal Health Insurance Database (LHID), a subset of the NHIRD which encompasses medical services provided to all insured individuals under the National Health Insurance (NHI) scheme. This includes 23 million Taiwanese citizens and covers disease diagnoses, examinations, drug prescriptions, outpatient and hospital visit codes, medical procedures, and charges. The NHI’s coverage rate has surpassed 99%, making NHIRD a significant source of empirical data for medical and health research. NHI, a compulsory and comprehensive health insurance program, has been in effect since 1995, covering over 23 million beneficiaries, which is more than 99% of the population, and is contracted with 97% of medical service providers. The program’s specifics have been detailed in numerous prior studies. NHIRD holds extensive data on outpatient and inpatient numbers. The inpatient dataset from 2000 to 2015, coded according to the International Classification of Diseases, Ninth Revision, Clinical Modification (ICD-9-CM), was selected for analysis.

NHIRD served as the primary data source for this study, standing as Taiwan’s most exhaustive healthcare research database. Diagnoses and procedures within NHIRD are classified following ICD-9-CM. The database is also connected to the National Death Registry and employs encryption of national identification numbers to safeguard individual privacy. Personal data within NHIRD is encrypted to maintain patient confidentiality. This study adhered to the Declaration of Helsinki and received approval from the Research Ethics Committee of the Tri-Service General Hospital, National Defense Medical College. (TSGHIRB: E202416046).

### Research design and participants

Starting January 1, 2000, NHI enrollees were included in the study. Exclusions were made for those with an MRDs prior to 2000, cardiomyopathy before tracking, drug disorders other than Amphetamine, no tracking, under 20 years of age, and unknown sex (*n* = 6,178). A diagnosis was established with at least one medical visit or hospitalization. Initially, individuals with an MRDs as of January 2000 were identified; subsequently, the rest were categorized as the unexposed group (non-MRDs) and matched in a 1:4 ratio by age, sex, and year of inclusion. The exposure date for the MRDs was set as the index date for the exposed group, with the same date applied to the matched subjects in the unexposed group. “Non-MRDs” refers to individuals not exposed to Methamphetamine (true control group). In February 2000, those selected for the study pairs and any individuals who had passed away in January were removed, and the same matching and indexing procedures were applied. This method was repeated monthly until the end of the observation period in December 2015.

To classify the three types of amphetamine use—dependence, abuse, and psychosis—it is essential to understand the diagnostic criteria and distinctions drawn from psychiatric and medical literature. These classifications align with diagnostic frameworks such as the DSM-5 (Diagnostic and Statistical Manual of Mental Disorders, 5th edition) and the ICD-10/ICD-11 (International Classification of Diseases).

1. Amphetamine and other psychostimulant dependence.

- This category falls under substance use disorder (SUD) in DSM-5 and corresponds to F15.2 (Mental and Behavioral Disorders due to Use of Other Stimulants: Dependence Syndrome) in ICD-10.
- Dependence is characterized by compulsive drug-seeking behavior, tolerance, withdrawal symptoms, and the inability to control use despite negative consequences.
- Research indicates that chronic and high-frequency use of amphetamines leads to neuroadaptive changes, reinforcing compulsive drug-seeking behaviors^16^.

2. Amphetamine or related acting sympathomimetic abuse.

- This category refers to harmful or problematic use that does not meet the criteria for dependence. In DSM-4, this was called Substance Abuse, but DSM-5 now includes it under the broader spectrum of SUD. In ICD-10, this aligns with F15.1 (Harmful Use of Stimulants).
- It typically includes episodic, recreational, or binge use that leads to short-term harm, such as cardiovascular effects, aggression, or risky behaviors.
- Studies show that intermittent, high-dose amphetamine use can still cause cognitive and psychiatric impairments without full-blown dependence^17^.

3. Amphetamine psychosis.

- Amphetamine-induced psychosis is recognized in both DSM-5 (Substance/Medication-Induced Psychotic Disorder) and ICD-10 (F15.5: Psychotic Disorder due to Stimulants).
- It involves symptoms like hallucinations, delusions, paranoia, and agitation, often resembling schizophrenia but directly triggered by amphetamine use.
- Research suggests that prolonged high dose use, or binge consumption significantly increases the risk of psychosis, but even short-term use in vulnerable individuals can trigger symptoms^18^.

#### Rationale for classification

The differentiation between these groups implies that:

- Dependence results from prolonged and frequent amphetamine use, leading to addiction.
- Abuse involves intermittent, non-dependent use that still causes harm.
- Psychosis can emerge independently of dependence, often linked to high doses or individual susceptibility.

The three types of MRDs—dependence, abuse, and psychosis—classify different levels and consequences of Methamphetamine use. Here’s how they relate to types of use and why distinguishing them is important:

#### Clarification of types of methamphetamine use

Each MRD category relates to different aspects of Methamphetamine use.

#### Mode of use

These categories do not directly classify how Methamphetamine is used (e.g., oral, snorted, injected, smoked), but different modes of use may increase the likelihood of certain disorders.

For example, injections and smoking have a higher risk of dependence and psychosis compared to oral use.

#### Length of use

Dependence usually results from chronic, long-term use.

Abuse can occur after short- or long-term use, depending on the pattern and severity.

Psychosis can emerge after both short- and long-term use, though chronic heavy use increases the risk.

#### Significance of comparing these codes

Clinical Implications—Helps determine the severity of the patient’s condition and the appropriate treatment approach.

Treatment & Intervention—Differentiating between abuse, dependence, and psychosis guides medical decisions, such as detox, rehabilitation, or antipsychotic treatment.

Legal & Insurance Considerations—Different diagnoses impact medical coding, insurance coverage, and legal outcomes (e.g., disability claims, criminal responsibility).

Research & Public Health—Understanding patterns of MRDs helps in tracking trends, designing prevention programs, and guiding policy decisions.

**Fig. 1:**
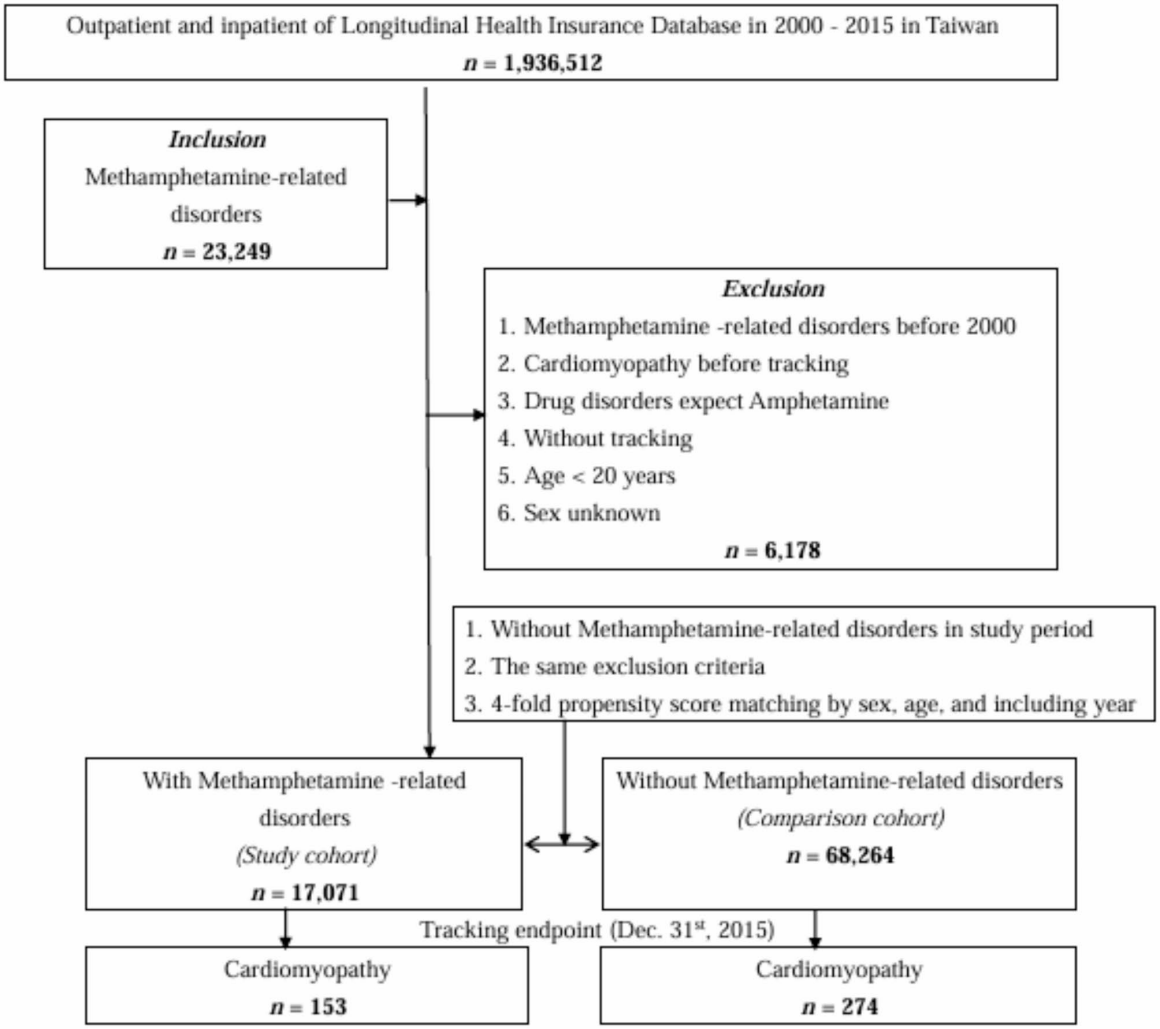
The flowchart of study sample selection.

A flowchart of the study design is depicted in Fig. 1.

### Covariates

Covariates included sociodemographic characteristics and comorbidities. Sociodemographic characteristics include sex, age (20–49, 50–64, ≧ 65 years old), monthly insurance premium, urbanization level, and area of residence. The monthly insurance premium has been divided into three categories in New Taiwan Dollars [NT$]: < 18,000, 18,000–34,999, and ≥ 35,000.

Season” refers to a specific time frame corresponding to the four calendar seasons: spring, summer, fall, and winter. For example, spring spans from March to May, summer from June to August, fall from September to November, and winter from December to February. Season was included as a demographic variable to explore potential temporal influences on the incidence and severity of methamphetamine-related cardiomyopathy (MRCM). This decision was based on evidence suggesting that seasonal factors—ranging from behavioral patterns of drug use to environmental stressors—may impact cardiovascular health. The rationale for including season as a variable in this study is threefold:

1. Seasonal variations in methamphetamine use: Previous research suggests that stimulant use, including methamphetamine, may fluctuate seasonally. Increased use has been reported in colder months, potentially due to higher rates of depression and social isolation, while summer months may see higher use in recreational settings such as festivals and nightlife^19^. If methamphetamine use follows seasonal patterns, fluctuations in exposure intensity could influence the incidence and severity of MRCM.
2. Winter-associated viral infections and myocardial injury: Viral infections, particularly influenza and other respiratory viruses, are more prevalent in winter and have been linked to increased cardiovascular morbidity, including myocarditis and myocardial dysfunction^20^. Given that methamphetamine exacerbates oxidative stress and cardiac inflammation, co-occurring viral infections could act as an additional trigger for myocardial injury, potentially worsening MRCM outcomes.
3. Environmental and physiological stressors: Cold weather increases sympathetic nervous system activity, vasoconstriction, and cardiac workload, leading to higher blood pressure and heart rate^21^. These physiological changes may compound methamphetamine’s known cardiotoxic effects, potentially leading to seasonal variations in MRCM severity.

The level of urbanization is the level of development of a city defined by population and certain indicators. The first-level urbanization rate is defined as a population exceeding 1,250,000 people. Secondary urbanization is defined as a population between 500,000 and 1,250,000. Urbanization levels 3 and 4 are defined as populations between 150,000 and 500,000 and less than 150,000, respectively. Charlson Comorbidity Index (CCI) is one of the most important widely used comorbidity index. The ICD codes for cardiomyopathy and comorbidities are listed in Table S1.

### Statistical analysis

The study utilized SPSS 22.0 software (SPSS Inc., Chicago, IL, USA) for statistical analysis, considering a *p*-value < 0.05 as statistically significant. Demographic characteristics and common comorbidities between patients with and without MRDs were compared using Pearson chi-square tests. Continuous variables were presented as means (± SD), and the mean age of patients was determined for two column pairs via a two-sample t-test. Age, sex, and concurrent comorbidities were adjusted for in the multivariable model analyses. Hazard ratios (HR) and 95% confidence intervals (CI) were derived from multivariate Cox proportional hazards models. The Kaplan-Meier method assessed the cardiomyopathy risk in patients with and without MRDs.

## Results

### Basic characteristics of patients in the study

Table 1 presents the demographic characteristics of patients with MRDs and their common comorbidities. From 2000 to 2015, the study included 17,071 patients with ARDs and 68,264 control subjects. The average age in the MRDs group was 43.82 ± 18.40 years; males constituted 70.25%, and females 29.75%. Significant differences between the MRDs group and controls were observed in monthly insurance premiums, CCI, residential area, urbanization level, and level of care, all with *p*-values less than 0.001.

**Table 1:** Characteristics of study in the baseline.

Methamphetamine-related disorders	Total		With		Without		*p*-value
Variables	*n*	%	*n*	%	*n*	%	
Total	85,355		17,071		68,284		
Sex							0.999
Male	59,960	70.25	11,992	70.25	47,968	70.25	
Female	25,395	29.75	5079	29.75	20,316	29.75	
Age (years)	44.04 ± 18.90		43.82 ± 18.40		44.09 ± 19.02		0.095
Age groups (yrs)							0.999
20–49	60,925	71.38	12,185	71.38	48,740	71.38	
50–64	8745	10.25	1749	10.25	6996	10.25	
≧ 65	15,685	18.38	3137	18.38	12,548	18.38	
Insured premium (NT$)							< 0.001
< 18,000	78,431	91.89	16,277	95.35	62,154	91.02	
18,000–34,999	4854	5.69	234	1.37	4620	6.77	
≧ 35,000	2070	2.43	560	3.28	1510	2.21	
CCI	1.00 ± 1.11		1.05 ± 1.24		0.99 ± 1.08		< 0.001
Season							0.999
Spring (Mar–May)	20,104	23.55	4022	23.56	16,082	23.55	
Summer (Jun–Aug)	22,592	26.47	4519	26.47	18,073	26.47	
Autumn (Sep–Nov)	21,624	25.33	4324	25.33	17,300	25.34	
Winter (Dec–Feb)	21,035	24.64	4206	24.64	16,829	24.65	
Location							< 0.001
Northern Taiwan	26,730	31.32	7378	43.22	19,352	28.34	
Middle Taiwan	22,420	26.27	4074	23.87	18,346	26.87	
Southern Taiwan	22,447	26.30	4612	27.02	17,835	26.12	
Eastern Taiwan	11,104	13.01	973	5.70	10,131	14.84	
Outlets islands	2654	3.11	34	0.20	2620	3.84	
Urbanization level							< 0.001
1 (The highest)	24,198	28.35	5997	35.13	18,201	26.65	
2 (Second)	30,215	35.40	7316	42.86	22,899	33.53	
3 (Third)	15,695	18.39	1320	7.73	14,375	21.05	
4 (The lowest)	15,247	17.86	2438	14.28	12,809	18.76	
Level of care							< 0.001
Hospital center	28,259	33.11	5495	32.19	22,764	33.34	
Regional hospital	33,353	39.08	9078	53.18	24,275	35.55	
Local hospital	23,743	27.82	2498	14.63	21,245	31.11	

P: Chi-square/Fisher exact test on category variables and t-test on continue variables.

### Characteristics of patient endpoints in the study

Table 2 shows the data of demographic characteristics for MRDs and associated comorbidities. From 2000 to 2015, the study included 17,071 MRDs patients and 68,264 controls. The MRDs group had an average age of 51.02 ± 18.97 years; males constituted 70.25%, while females accounted for 29.75%. Significant differences between the study and control groups included cardiomyopathy, age, age groups, monthly insurance premiums, CCI, season, residential area, urbanization level, and level of care, all with *p*-values less than 0.001.

**Table 2:** Characteristics of study in the endpoint.

Methamphetamine-related disorders	Total		With		Without		*p*-value
Variables	*n*	%	*n*	%	*n*	%	
Total	85,355		17,071		68,284		
Cardiomyopathy							< 0.001
Without	84,928	99.50	16,918	99.10	68,010	99.60	
With	427	0.50	153	0.90	274	0.40	
Sex							0.999
Male	59,960	70.25	11,992	70.25	47,968	70.25	
Female	25,395	29.75	5079	29.75	20,316	29.75	
Age (yrs)	53.28 ± 19.76		51.02 ± 18.97		53.84 ± 19.91		< 0.001
Age groups (yrs)							< 0.001
20–49	50,488	59.15	11,060	64.79	39,428	57.74	
50–64	14,575	17.08	2099	12.30	12,476	18.27	
≧ 65	20,292	23.77	3912	22.92	16,380	23.99	
Insured premium (NT$)							< 0.001
< 18,000	78,431	91.89	16,277	95.35	62,154	91.02	
18,000–34,999	4854	5.69	234	1.37	4620	6.77	
≧ 35,000	2070	2.43	560	3.28	1510	2.21	
CCI	1.03 ± 1.33		1.10 ± 1.29		1.01 ± 1.09		< 0.001
Season							< 0.001
Spring	19,042	22.31	3745	21.94	15,297	22.40	
Summer	24,615	28.84	4631	27.13	19,984	29.27	
Autumn	21,009	24.61	4289	25.12	16,720	24.49	
Winter	20,689	24.24	4406	25.81	16,283	23.85	
Location							< 0.001
Northern Taiwan	25,524	29.90	7142	41.84	18,382	26.92	
Middle Taiwan	22,079	25.87	4023	23.57	18,056	26.44	
Southern Taiwan	23,511	27.54	4539	26.59	18,972	27.78	
Eastern Taiwan	13,116	15.37	1012	5.93	12,104	17.73	
Outlets islands	1125	1.32	355	2.08	770	1.13	
Urbanization level							< 0.001
1 (The highest)	23,972	28.09	5986	35.07	17,986	26.34	
2 (Second)	29,587	34.66	6483	37.98	23,104	33.84	
3 (Third)	14,737	17.27	1978	11.59	12,759	18.69	
4 (The lowest)	17,059	19.99	2624	15.37	14,435	21.14	
Level of care							< 0.001
Hospital center	26,995	31.63	5322	31.18	21,673	31.74	
Regional hospital	31,650	37.08	8249	48.32	23,401	34.27	
Local hospital	26,710	31.29	3500	20.50	23,210	33.99	

P: Chi-square/Fisher exact test on category variables and t-test on continue variables.

### Years from methamphetamine-related disorders to cardiomyopathy

The mean time after diagnosis from the index date to the diagnosis of cardiomyopathy was 10.24 (SD = 9.86) years. Patients with MRDs developed cardiomyopathy at a mean age of 5.62 (SD = 3.89) years earlier than patients without MRDs (6.21 [SD = 4.20] years) (Table S2-1, Table S2-2).

### Use Cox regression to analyze the influencing factors of cardiomyopathy in methamphetamine-related disorders

Table 3 presents the results of the cox regression analysis on risk factors within the MRDs study cohort and the comparison group. The study reveals that after adjusting for sex, age, insurance premiums, CCI, season, location, urbanization level, and level of care, individuals with MRDs have a 3.421 times higher risk of developing cardiomyopathy compared to non-MRDs. Gender-wise, men have a 0.735 times lower risk of developing cardiomyopathy than women. Age-wise, individuals aged 50–64 and those 65 or older have a 1.145- and 1.332-times higher risk, respectively, compared to those aged 20–49. For each one-point increase in CCI, the risk of cardiomyopathy rises by 58.3%. Seasonally, the risk in winter is 1.672 times higher than in spring. Regarding urbanization, the first and second levels have a 2.210- and 1.563-times higher risk, respectively, compared to the fourth level. Finally, concerning the level of care, hospital centers and regional hospitals have a 1.985- and 1.301-times higher risk, respectively, compared to local hospitals.

**Table 3:** Factors of cardiomyopathy by using Cox regression.

Variables	Adjusted HR	95% CI	*p*-value
Methamphetamine-related disorders			
Without	Reference		
With	3.421	1.589–5.014	< 0.001
Sex			
Male	0.735	0.579–0.902	0.001
Female	Reference		
Age group (yrs)			
20–49	Reference		
50–64	1.145	1.080–1.279	0.010
≧ 65	1.332	1.107–1.522	< 0.001
Insured premium (NT$)			
< 18,000	Reference		
18,000–34,999	1.005	0.553–1.184	0.449
≧ 35,000	1.123	0.742–1.376	0.255
CCI	1.583	1.108–1.910	< 0.001
Season			
Spring	Reference		
Summer	1.240	0.682–1.805	0.317
Autumn	1.045	0.498–1.633	0.502
Winter	1.672	1.010–2.249	0.045
Location	Multicollinearity with urbanization level		
Northern Taiwan	Multicollinearity with urbanization level		
Middle Taiwan	Multicollinearity with urbanization level		
Southern Taiwan	Multicollinearity with urbanization level		
Eastern Taiwan	Multicollinearity with urbanization level		
Outlets islands	Multicollinearity with urbanization level		
Urbanization level			
1 (The highest)	2.210	1.865–2.511	< 0.001
2 (Second)	1.563	1.101–1.845	< 0.001
3 (Third)	1.121	0.842–1.497	0.157
4 (The lowest)	Reference		
Level of care			
Hospital center	1.985	1.484–2.136	< 0.001
Regional hospital	1.301	1.112–1.573	< 0.001
Local hospital	Reference		

HR = hazard ratio, CI = confidence interval, Adjusted HR: Adjusted variables listed in the table.

### Methamphetamine—related disorders using Cox regression to stratify cardiomyopathy factors by the variables listed in the table

Table 4 presents the incidence and hazard ratio (HR) of cardiomyopathy among individuals with and without MRDs relative to controls. Accounting for variables such as sex, age, insurance premiums, CCI, season, location, urbanization level, and level of care, the study found that patients with MRDs had a 3.421-fold higher incidence of cardiomyopathy compared to those without MRDs (*p* < 0.001). Gender-wise, male MRDs patients had a 3.178-fold higher HR for cardiomyopathy than non-MRDs patients (*p* < 0.001), while female ARDs patients had a 4.011-fold increase (*p* < 0.001). Age-wise, MRDs patients aged 20–49 had a 2.978-fold higher risk, those 50–64 had a 3.402-fold increase, and those aged 65 and above had a 4.551-fold higher risk of cardiomyopathy compared to non-MRDs patients (*p* < 0.001).

**Table 4:** Factors of cardiomyopathy stratified by variables listed in the table by using Cox regression and bonferroni correction for multiple comparisons.

Methamphetamine—related disorders	With			Without (Reference)			With vs. without (Reference)			
Stratified	Events	PYs	Rate	Events	PYs	Rate	aHR	95% CI		*p*-value
Total	153	198,273.14	77.17	274	793,065.24	34.55	3.421	1.589	5.014	< 0.001
Sex										
Male	101	139,782.56	72.26	194	557,110.79	34.82	3.178	1.474	4.659	< 0.001
Female	52	58,490.58	88.90	80	235,954.45	33.90	4.011	1.862	5.888	< 0.001
Age group (yrs)										
20–49	78	128,457.63	60.72	142	457,925.30	31.01	2.978	1.396	4.384	< 0.001
50–64	21	24,379.81	86.14	56	144,898.36	38.65	3.402	1.587	5.013	< 0.001
≧ 65	54	45,435.70	118.85	76	190,241.58	39.95	4.551	2.110	6.682	< 0.001
Insured premium (NT$)										
< 18,000	131	189,052.24	69.29	240	721,780.11	33.25	3.101	1.472	4.683	< 0.001
18,000–34,999	3	2,789.33	107.55	19	53,657.59	35.41	4.248	2.159	6.580	< 0.001
≧ 35,000	19	6,431.57	295.42	15	17,627.54	85.09	5.303	2.471	7.774	< 0.001
Season										
Spring	32	43,498.25	73.57	60	177,666.14	33.77	3.333	1.551	4.897	< 0.001
Summer	43	53,782.11	79.95	81	232,098.25	34.90	3.522	1.638	5.149	< 0.001
Autumn	30	49,818.66	60.22	58	194,186.31	29.87	3.024	1.421	4.505	< 0.001
Winter	48	51,174.12	93.80	75	189,114.54	39.66	3.622	1.689	5.311	< 0.001
Urbanization level										
1 (The highest)	59	69,525.04	84.86	75	208,893.35	35.90	3.611	1.690	5.303	< 0.001
2 (Second)	61	75,297.63	81.01	93	268,334.42	34.66	3.572	1.667	5.247	< 0.001
3 (Third)	18	22,973.71	78.35	51	148,185.80	34.42	3.485	1.624	5.111	< 0.001
4 (The lowest)	15	30,476.76	49.22	55	167,651.67	32.81	2.291	1.065	3.368	0.018
Level of care										
Hospital center	51	61,818.24	82.50	89	251,716.87	35.36	3.578	1.662	5.270	< 0.001
Regional hospital	75	95,809.17	78.28	94	271,784.30	34.59	3.432	1.598	5.011	< 0.001
Local hospital	27	40,645.73	66.43	91	269,564.07	33.76	3.012	1.325	4.106	< 0.001

PYs = Person-years; Rate: per 10^5^ PYs; AHR = Adjusted Hazard ratio: Adjusted for the variables listed in Table 3; CI = confidence interval.

### Factors of cardiomyopathy across various subgroups of methamphetamine e-related disorders using Cox regression and applying bonferroni correction for multiple comparisons

Table 5 presents the incidence and hazard ratio (HR) of cardiomyopathy among individuals with and without MRDs subgroups relative to controls. Accounting for variables such as sex, age, insurance premiums, CCI, season, location, urbanization level, and level of care, the study found that the incidence of cardiomyopathy was 0.77 cases per 100,000 person-years (PYs) in patients with MRDs, compared to 0.34 cases per 100,000 PYs in those without MRDs. The HR for cardiomyopathy was 3.421 times higher in patients with MRDs than in those without (*p* < 0.001). Specifically, for three types of Methamphetamines (Methamphetamine and other psychostimulant dependence, Methamphetamine or related acting sympathomimetic abuse, Methamphetamine psychosis), the HR for cardiomyopathy in patients with MRDs was 3.864 (*p* < 0.001), 2.916 (*p* < 0.001), and 2.295 (*p* = 0.016) times higher, respectively, compared to patients without MRDs.

**Table 5:** Factors of cardiomyopathy among different methamphetamine-related disorders subgroups by using Cox regression and bonferroni correction for multiple comparisons.

Methamphetamine-related disorders	Populations	Events	PYs	Rate	aHR	95% CI	*p*-value
Without	68,284	274	793,065.24	34.55	Reference		
With	17,071	153	198,273.14	77.17	3.421	1.589–5.014	< 0.001
Methamphetamine and other psychostimulant dependence	11,254	111	130,718.94	84.92	3.864	1.802–5.522	< 0.001
Methamphetamine or related acting sympathomimetic abuse	4321	33	50,189.22	65.75	2.916	1.358–4.271	< 0.001
Methamphetamine psychosis	1496	9	17,364.98	51.83	2.295	1.068–3.360	0.016

PYs = Person-years; Rate: per 10^5^ PYs; AHR = Adjusted Hazard ratio: Adjusted for the variables listed in Table 3.; CI = confidence interval.

### Kaplan-Meier cumulative incidence curve of cardiomyopathy in methamphetamine-related disorders

**Fig. 2:**
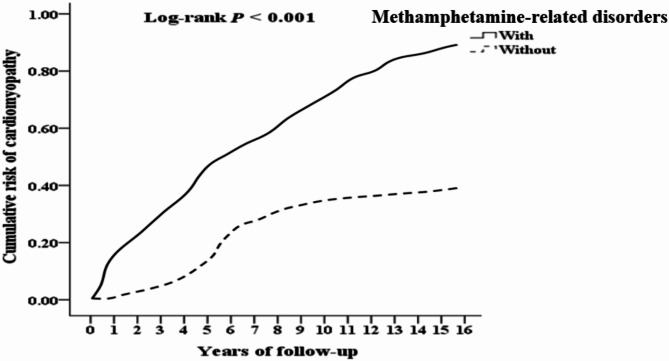
Kaplan-Meier for cumulative risk of cardiomyopathy aged 20 and over stratified by Methamphetamine-related disorders with log-rank test.

Figure 2 reveals the cumulative incidence of cardiomyopathy (long-rank test, *p* < 0.001) in the MRDs group (*n* = 17,071) and the non-MRDs group (*n* = 68,264) during follow-up. There was a significant difference in the cumulative incidence of cardiomyopathy between the MRDs group and the non-MRDs group (long-rank test, *p* < 0.001).

## Discussion

This population-based cohort study showed that patients with MRDs had a 3.421-folds higher risk of cardiomyopathy than patients without MRDs. Men have a 0.735-fold lower risk of developing cardiomyopathy than women. In terms of age group, aged 50–64 and ≧ 65 have a 1.145- and 1.332-folds higher risk of cardiomyopathy, respectively, compared to those aged 20–49. For each one-point increase in CCI, the risk of cardiomyopathy rises by 58.3%. Specifically, for three types of Methamphetamines (Methamphetamine and other psychostimulant dependence, Methamphetamine or related acting sympathomimetic abuse, Methamphetamine psychosis), the HR for cardiomyopathy in patients with MRDs was 3.864 (*p* < 0.001), 2.916 (*p* < 0.001), and 2.295 (*p* = 0.016) times higher, respectively, compared to patients without MRDs. The Kaplan-Meier log-rank test was used to calculate the cumulative risk of MRDs, showing a significant difference in the cumulative cardiomyopathy incidence between the MRDs and non-MRDs groups (long-rank test, *p* < 0.001).

Methamphetamine-induced cardiomyopathy, a rare yet serious heart condition, arises from prolonged Methamphetamine use. Journal of Cardiology Cases reported on a 37-year-old individual who suffered from dilated cardiomyopathy as a result of chronic Methamphetamine consumption^22^. Another published case report describes a patient with severe Methamphetamine-induced cardiomyopathy who was successfully treated with heart transplantation. This study re-emphasizes the critical role of early intervention and appropriate management in improving patient outcomes^23^. Although the exact mechanism of Methamphetamine-induced cardiomyopathy is not fully understood, several possible pathways have been proposed, including oxidative stress, mitochondrial dysfunction, and neurohormonal priming^24^. Methamphetamine use causes cardiomyopathy through catecholamine-mediated effects such as tachycardia, hypertension, vasoconstriction and direct cardiotoxic effects^25^. Stimulants such as Methamphetamine exert negative effects through catecholamines, causing tachycardia, hypertension, vasoconstriction, and vasospasm^26,27^. Sustained exposures to catecholamines can lead to cardiotoxic effects, including changes in myocardial contractility and myocardial fibrosis^28,29^. Structural changes may include dilated cardiomyopathy, with or without reduced ejection fraction, or hypertrophic cardiomyopathy^30^. Emerging evidence suggests that acute Epstein-Barr virus (EBV) infection, identified through virological screening, may contribute to the development of cardiomyopathy, particularly in individuals with a history of Methamphetamine use. This highlights a potential interplay between viral myocarditis and Methamphetamine-induced cardiotoxicity, warranting further investigation into their combined effects on myocardial function^25^. Recent studies indicate that the incidence of Methamphetamine-induced cardiomyopathy appears to be increasing, possibly due to increased availability and use of Methamphetamines, particularly in younger adults. Additionally, there is growing evidence that the combined use of Methamphetamines with other drugs, such as heroin or opioids, may further increase the risk of cardiomyopathy^8^. Some studies suggest that early identification and cessation of Methamphetamine use may improve outcomes, while others find that long-term prognosis for these patients remains poor even with aggressive drug treatment^31,32^.

Comparison of clinical features and outcomes of patients with reversible and persistent methamphetamine-associated cardiomyopathy showed that MAC reversal is not uncommon and is associated with significant clinical improvement including reduced mortality. It can be facilitated by MA cessation when the cardiac chambers, especially the right ventricle, are not severely dilated^33–41^. Clinical correlates and outcomes of methamphetamine-associated cardiovascular disease among hospitalized patients in California; methamphetamine use was associated with a similar magnitude of CVD risk compared with alcohol and heroin^42^. The CCI is associated with the risk of 30-day mortality in patients with myocardial injury after non-cardiac surgery^43^. Studies have identified three types of Methamphetamine use—dependence, abuse, and psychosis—as being associated with an increased hazard ratio (HR) for cardiomyopathy^44,45^. The distinction between these three types of Methamphetamine use—dependence, abuse, and psychosis—suggests important differences in the duration, frequency, and severity of use.

1. Methamphetamine and other psychostimulant dependence: This category refers to chronic and compulsive use, where individuals develop tolerance, experience withdrawal symptoms, and prioritize drug use over other aspects of life. Dependence typically implies long-term, high-frequency exposure to Methamphetamines.
2. Methamphetamine or related acting sympathomimetic abuse: This classification includes sporadic or less frequent misuse that does not necessarily lead to full dependence but still results in harmful consequences. It suggests that individuals in this group engage in episodic or recreational use, potentially with lower cumulative exposure compared to dependent users.
3. Methamphetamine psychosis: This refers to a state where amphetamine use leads to psychotic symptoms, such as hallucinations or paranoia. While psychosis can occur in both short- and long-term users, it is more commonly associated with high doses, chronic use, or binge patterns. However, some individuals may develop psychosis even with relatively short-term but intense use.

The distinction between these groups implies that longer exposure and more frequent use increases the risk of dependence and psychosis, but individual susceptibility, dosage, and patterns of use also play key roles. The above research results are similar to our research results.

In Taiwan, the majority of amphetamines abused are methamphetamine, which accounts for 40% of illicit drug use, second only to heroin^46^. Additionally, Methamphetamine drugs (such as dextroamphetamine) are not licensed or reimbursed by Taiwan’s NHI program. Additionally, people who had poor impulse control in childhood may be prone to abusing methamphetamine because its effects occur more quickly and last longer than amphetamine drugs [47.48]. The long-term prognosis of patients with Methamphetamine-induced cardiomyopathy remains poor, highlighting the need for increased awareness and prevention efforts around this growing public health problem^8^. “Our findings align with global trends, as demonstrated by Raja et al. (2024), who reported an increase in substance-induced cardiomyopathy-related mortality among older adults in the United States from 1999 to 2020^49^. This reinforces the need for early cardiovascular screening in individuals with MRDs.”

### Strengths

Currently, there is limited research on the relationship between MRDs and its impact on the development of cardiomyopathy in the general population. To our knowledge, our study is the largest and longest nationwide cohort study investigating the association between MRDs and cardiomyopathy risk in Taiwan from 2000 to 2015. Our study shows that people with MRDs have a higher risk of cardiomyopathy than people without MRDs, women have a greater risk of cardiomyopathy than men, those aged 50–64 and ≧ 65 years have a greater risk of injury than those aged 20–49 years. For each one-point increase in CCI, the risk of cardiomyopathy rises by 58.3%. Specifically, for three types of Methamphetamines (Methamphetamine and other psychostimulant dependence, Methamphetamine or related acting sympathomimetic abuse, Methamphetamine psychosis), the HR for cardiomyopathy in patients with MRDs was higher compared to patients without MRDs. Kaplan-Meier showed a significant difference in the incidence of cumulative cardiomyopathy between the MRDs and non-MRDs groups. Distinctions between the three types of Methamphetamine use (dependence, abuse, and psychosis) suggest that there are important differences between these groups in terms of duration, frequency, and severity of use, with longer exposure and more frequent use increasing the risk of dependence and psychosis, but individual susceptibility, dose, and use patterns also play key roles.

### Limitations

This study has some limitations. First, this is a generational follow-up study based on the Taiwanese population, so our findings may not be generalizable to other regions and ethnic groups. Second, we were unable to assess the lifestyle, behavioural patterns, alcohol consumption, genetic factors, psychosocial factors, environmental factors, severity, or psychological assessment of patients with MRDs because these data were not recorded in the NHI research repository. Third, we used data from the NHIRD rather than self-reports from patients with MRDs. Therefore, the prevalence of MRDs or the incidence of cardiomyopathy may still be underestimated because the database only contains data on those who seek medical care. There were no reported cases of patients with MRDs who did not present to clinics or emergency rooms, which may have increased bias in the study. Finally, we were unable to account for the onset and duration of comorbidities, which may influence changes in MRDs.

## Conclusion

The study indicates that individuals with MRDs have a significantly higher risk of developing cardiomyopathy compared to those without MRDs. It also suggests that women are more susceptible to cardiomyopathy than men, and the risk escalates for individuals aged 50–64 and those 65 years or older, compared to the 20–49-year age group. Additionally, an increase in the CCI correlates with a heightened risk of cardiomyopathy. The study found that HR for cardiomyopathy is elevated in cases of three types of MRDs: dependence on Methamphetamines and other psychostimulants, abuse of Methamphetamines or related sympathomimetics, and Methamphetamine psychosis. These findings could inform the creation of cardiomyopathy prevention strategies. There are important differences between these groups in terms of duration, frequency, and severity of use, with longer exposure and more frequent use increasing the risk of dependence and psychosis, but individual susceptibility, dose, and use patterns also play key roles.

## Electronic supplementary material

Below is the link to the electronic supplementary material.

Supplementary Material 1

## Data Availability

The data that support the findings of this study are available from the corresponding author upon reasonable request.
