# Supplementary material for: Increased risk of cardiomyopathy in individuals with methamphetamine related disorders in Taiwan
Source: Sci Rep. 2025 Apr 3;15:11449. doi: 10.1038/s41598-025-94591-0 (PMC11968872; doi:10.1038/s41598-025-94591-0)
Supplement: Supplementary file 1 — Supplementary Material 1 [file 41598_2025_94591_MOESM1_ESM.docx]

Supplementary Materials

Table S1. Abbreviation, ICD-9-CM, and definition

|  | Abbreviation | ICD-9-CM / Definition |
| --- | --- | --- |
| Study population: Amphetamine-related disorders |  |  |
| Amphetamine and other psychostimulant dependence |  | 304.4 |
| Amphetamine or related acting sympathomimetic abuse |  | 350.7 |
| Amphetamine psychosis |  | 292; between Jan 1^st^, 2000, and Dec 31^st^, 2000 |
| Excluding: Drug disorders |  | 304.0 - 304.3, 304.5 - 304.9, 350.0 - 350.6, 350.8, 350.9; 1-year previous and within 1-year of 292 diagnosis |
| Events: Cardiomyopathy |  | 425 |
| Comorbidities: Charlson comorbidity index | CCI |  |

Table S2-1. Years of follow-up

| Amphetamine-related disorders | Min | Median | Max | Mean ± SD | *p*-value |
| --- | --- | --- | --- | --- | --- |
| With | 0.01 | 7.82 | 15.90 | 10.24 ± 9.86 |  |
| Without | 0.01 | 7.97 | 15.92 | 10.45 ± 9.95 |  |
| Total | 0.01 | 7.86 | 15.92 | 10.41 ± 9.93 | 0.014 |

Table S2-2. Years to cardiomyopathy

| Amphetamine-related disorders | Min | Median | Max | Mean ± SD | *p*-value |
| --- | --- | --- | --- | --- | --- |
| With | 0.03 | 4.23 | 15.24 | 5.62 ± 3.89 |  |
| Without | 0.04 | 5.92 | 15.76 | 6.21 ± 4.20 |  |
| Total | 0.03 | 4.91 | 15.76 | 6.09 ± 4.15 | < 0.001 |

Table S3. Factors of cardiomyopathy by using Cox regression (Adjusted for comorbidities)

| Adjusted for comorbidities | *Model 1: Adjusted for CCI* | | | *Model 2: Adjusted for cardiovascular diseases* | | | *Model 3: Adjusted for cardiovascular diseases and CCI_R* | | |
| --- | --- | --- | --- | --- | --- | --- | --- | --- | --- |
| Variables | aHR | 95% CI | *p*-value | aHR | 95% CI | *p*-value | aHR | 95% CI | *p*-value |
| Amphetamine-related disorders | | | | | | | | | |
| Without | Reference |  |  | Reference |  |  | Reference |  |  |
| With | 3.421 | 1.589  5.014 | < 0.001 | 2.897 | 1.432  4.020 | < 0.001 | 2.330 | 1.279  3.865 | < 0.001 |
| Sex | | | | | | | | | |
| Male | 0.735 | 0.579  0.902 | 0.001 | 0.686 | 0.468  0.838 | < 0.001 | 0.712 | 0.539  0.899 | < 0.001 |
| Female | Reference |  |  | Reference |  |  | Reference |  |  |
| Age group (yrs) | | | | | | | | | |
| 20 - 49 | Reference |  |  | Reference |  |  | Reference |  |  |
| 50 - 64 | 1.145 | 1.080  1.279 | 0.010 | 1.159 | 1.090  1.285 | 0.005 | 1.150 | 1.082  1.273 | 0.008 |
| ≧ 65 | 1.332 | 1.107  1.522 | < 0.001 | 1.346 | 1.111  1.523 | < 0.001 | 1.326 | 1.104  1.518 | < 0.001 |
| Insured premium (NT$) | | | | | | | | | |
| < 18,000 | Reference |  |  | Reference |  |  | Reference |  |  |
| 18,000 - 34,999 | 1.005 | 0.553  1.184 | 0.449 | 1.004 | 0.552  1.173 | 0.432 | 1.006 | 0.555  1.193 | 0.483 |
| ≧ 35,000 | 1.123 | 0.742  1.376 | 0.255 | 1.121 | 0.740  1.365 | 0.251 | 1.129 | 0.748  1.396 | 0.267 |
| DM | | | | | | | | | |
| Without |  |  |  | Reference |  |  | Reference |  |  |
| With |  |  |  | 1.597 | 1.148  1.986 | < 0.001 | 1.523 | 1.121  1.973 | < 0.001 |
| Hyperlipidemia | | | | | | | | | |
| Without |  |  |  | Reference |  |  | Reference |  |  |
| With |  |  |  | 1.325 | 1.020  1.572 | 0.040 | 1.301 | 1.014  1.522 | 0.012 |
| HTN | | | | | | | | | |
| Without |  |  |  | Reference |  |  | Reference |  |  |
| With |  |  |  | 2.567 | 1.335  4.443 | < 0.001 | 2.432 | 1.320  4.328 | < 0.001 |
| IHD | | | | | | | | | |
| Without |  |  |  | Reference |  |  | Reference |  |  |
| With |  |  |  | 2.371 | 1.268  4.252 | < 0.001 | 2.270 | 1.245  4.136 | < 0.001 |
| AF | | | | | | | | | |
| Without |  |  |  | Reference |  |  | Reference |  |  |
| With |  |  |  | 1.568 | 1.124  1.788 | < 0.001 | 1.553 | 1.102  1.770 | < 0.001 |
| HF | | | | | | | | | |
| Without |  |  |  | Reference |  |  | Reference |  |  |
| With |  |  |  | 1.333 | 1.042  1.573 | 0.029 | 1.324 | 1.031  1.559 | 0.035 |
| CVD | | | | | | | | | |
| Without |  |  |  | Reference |  |  | Reference |  |  |
| With |  |  |  | 1.865 | 1.567  2.371 | < 0.001 | 1.765 | 1.560  2.274 | < 0.001 |
| PAD | | | | | | | | | |
| Without |  |  |  | Reference |  |  | Reference |  |  |
| With |  |  |  | 1.722 | 1.432  1.989 | < 0.001 | 1.666 | 1.389  1.975 | < 0.001 |
| CCI | 1.583 | 1.108  1.910 | < 0.001 |  |  |  |  |  |  |
| CCI_R |  |  |  |  |  |  | 1.577 | 1.353  1.762 | < 0.001 |
| Season | | | | | | | | | |
| Spring | Reference |  |  | Reference |  |  | Reference |  |  |
| Summer | 1.240 | 0.682  1.805 | 0.317 | 1.135 | 0.656  1.798 | 0.335 | 1.139 | 0.762  1.776 | 0.333 |
| Autumn | 1.045 | 0.498  1.633 | 0.502 | 1.059 | 0.562  1.637 | 0.513 | 1.060 | 0.465  1.656 | 0.598 |
| Winter | 1.672 | 1.010  2.249 | 0.045 | 1.633 | 0.986  2.235 | 0.072 | 1.640 | 1.002  2.353 | 0.048 |
| Urbanization level | | | | | | | | | |
| 1 (The highest) | 2.210 | 1.865  2.511 | < 0.001 | 2.211 | 1.986  2.532 | < 0.001 | 2.204 | 1.957  2.434 | < 0.001 |
| 2 | 1.563 | 1.101  1.845 | < 0.001 | 1.578 | 1.234  1.901 | < 0.001 | 1.567 | 1.222  1.894 | < 0.001 |
| 3 | 1.121 | 0.842  1.497 | 0.157 | 1.133 | 0.886  1.532 | 0.114 | 1.131 | 0.880  1.524 | 0.120 |
| 4 (The lowest) | Reference |  |  | Reference |  |  | Reference |  |  |
| Level of care | | | | | | | | | |
| Hospital center | 1.985 | 1.484  2.136 | < 0.001 | 2.012 | 1.584  2.213 | < 0.001 | 2.008 | 1.559  2.121 | < 0.001 |
| Regional hospital | 1.301 | 1.112  1.573 | < 0.001 | 1.456 | 1.134  1.602 | < 0.001 | 1.444 | 1.121  1.583 | < 0.001 |
| Local hospital | Reference |  |  | Reference |  |  | Reference |  |  |

Model 1 (CCI), Model 2 (DM, hyperlipidemia, HTN, IHD, AF, HF, CVD, PAD), Model 3 (8 comorbidities + CCI_R)

HR= hazard ratio, CI = confidence interval, aHR = Adjusted HR: Adjusted variables listed in the table; Location had
